# CT Image Segmentation Using FEM with Optimized Boundary Condition

**DOI:** 10.1371/journal.pone.0031116

**Published:** 2012-02-28

**Authors:** Hiroyuki Hishida, Hiromasa Suzuki, Takashi Michikawa, Yutaka Ohtake, Satoshi Oota

**Affiliations:** 1 Department of Precision Engineering, School of Engineering, The University of Tokyo, Bunkyo-ku, Tokyo, Japan; 2 Research Center for Advanced Science and Technology, The University of Tokyo, Meguro-ku, Tokyo, Japan; 3 RIKEN BioResouce Center, Tsukuba, Ibaraki, Japan; University of California, Berkeley, United States of America

## Abstract

The authors propose a CT image segmentation method using structural analysis that is useful for objects with structural dynamic characteristics. Motivation of our research is from the area of genetic activity. In order to reveal the roles of genes, it is necessary to create mutant mice and measure differences among them by scanning their skeletons with an X-ray CT scanner. The CT image needs to be manually segmented into pieces of the bones. It is a very time consuming to manually segment many mutant mouse models in order to reveal the roles of genes. It is desirable to make this segmentation procedure automatic. Although numerous papers in the past have proposed segmentation techniques, no general segmentation method for skeletons of living creatures has been established. Against this background, the authors propose a segmentation method based on the concept of destruction analogy. To realize this concept, structural analysis is performed using the finite element method (FEM), as structurally weak areas can be expected to break under conditions of stress. The contribution of the method is its novelty, as no studies have so far used structural analysis for image segmentation. The method's implementation involves three steps. First, finite elements are created directly from the pixels of a CT image, and then candidates are also selected in areas where segmentation is thought to be appropriate. The second step involves destruction analogy to find a single candidate with high strain chosen as the segmentation target. The boundary conditions for FEM are also set automatically. Then, destruction analogy is implemented by replacing pixels with high strain as background ones, and this process is iterated until object is decomposed into two parts. Here, CT image segmentation is demonstrated using various types of CT imagery.

## Introduction

We propose a CT image segmentation method using structural analysis that is useful for objects with structural dynamic characteristics. Motivation of our research is from the area of genetic activity. In order to reveal the roles of genes, it is necessary to create mutant mice and measure differences among them, in particular, morphological difference in their skeletons. X-ray CT is used to scan a skeleton then its image is manually segmented into pieces of the bones. Considering the number of genes involved, it is desirable to make this segmentation procedure automatic. A problem specific to this segmentation is to separate bones at their joints where the gap between the bones are not necessarily clear because of their complex tissue structures. Despite the numerous image segmentation methods, none of them matches our objectives.

Accordingly, we previously proposed a method to segment CT images using structural analysis [1]. The technique is based on the assumption that the interference area (joint) between components (bones) is structurally weak. We compute strain, which tends to be large in structurally weak areas and segment the image in the region of high strain. In previous work [1], we used a commercial software VOXELCON [2], which is an image-based structural analysis system. In the approach examined, we set physical properties for every pixel and create a stiffness matrix. Then, we calculated the von Mises strain of every pixel and removed the one with the highest strain value, assuming it will be broken. We iterated strain calculation and removal until the input image is decomposed into multiple parts. The method could be used to segment low-contrast CT images - a task that was difficult with conventional methods. This technique was based on the use of structural analysis for CT image segmentation. However, it was necessary with this approach to set boundary conditions manually in structural analysis. Thus trial and error is required to apply the method.

In this paper, we expand our previous work [1] to set parameters and loading conditions in a semiautomatic way. We set regions of interest (ROIs) as candidates for weak areas, which include correct and erroneous segmentation targets. Assuming strain as an objective function, we calculate boundary conditions for each ROI using a gradient ascent method [3]. In other words, we calculate strain for all ROIs. Then, we choose the ROI with the highest strain as the correct candidate for a weak area, and these conditions are then used to segment the image.

The main advantage of the proposed approach is its reliability, as the structural analysis it involves means that appropriate object segmentation in structurally weak areas is guaranteed. As this method is designed to segment joints of a skeleton and thus prefers such objects with shapes of relatively high aspect ratio around the segmentation boundaries. The originality of this research lies in its introduction of the analogy of mechanical destruction to image processing and its optimization of boundary conditions in the finite element method (FEM).

### Related work

Numerous papers on image segmentation have been published. Most image segmentation is performed using image processing technology, but the proposed approach stands apart from conventional methods because we introduce the concept of structural analysis.

Thresholding is the simplest method of segmentation, and involves the assumption that CT values are related to an object's density. Adaptive thresholding is used in [4], but it requires parameter tuning. It is also difficult for the method to produce error-free results, since the CT values of pixels are influenced by the density of neighboring areas.

Kass et al. [5] proposed the snakes algorithm, which is an active contour method that minimizes spline energy for segmentation. This approach assumes that the target object is smooth and has strong edges. As the snakes algorithm approach is a powerful tool for CT image segmentation, there are many papers on it, such as [6], [7]. However, if the edges of bones are weak or the distance between bones is minimal, the approach fails in image segmentation.

Adams et al. [8] proposed the seeded region growing method. This technique involves propagating an area of given seeds using pixel values and distances. Although the application of region growing to image segmentation has been widely reported, such as [9], [10], and problems remain with regard to the difficulty of selecting the right seeds.

Vincent et al. [11] introduced the watershed approach, which uses pixel gradient values to extract regions in the same way as water flooding. The method assumes that neighboring pixels with similar values should be labeled as parts of the same cluster. It is a powerful technique on which many works have been published, such as [12], [13]. However this approach has the disadvantage of over-segmentation, and also we cannot apply a cost function optimized for one dataset to other datasets, as objects in biological application are variable and not uniform.

Boykov et al. [14] introduced the graph-cut algorithm, which uses the max-flow min-cut theorem [15] to segment graph edges. In this approach, the cost function is set appropriately from the target in graph representation. Since graph cut is a powerful method, many related papers have been published, such as [16], [17]. [17] includes a survey on segmentation for parts of living things. However, it is not easy to set a cost function appropriately and with high versatility, and a cost function optimized for one dataset cannot be applied to other datasets, as with the watershed method.

FEM is used in image processing in different contexts. First of all, in the field of bio-mechanics it is a common approach to apply FEM analysis with CT or MR images [18], [19]. In the most cases they use scanned images for creating mesh models for applying 3D FEM analysis and the image segmentation was done with some conventional methods. Recently, Auer and Gasser [20] proposed FEM-based deformable models to reconstruct aneurysms from CT images. It can be considered as an active contour based segmentation. The difference from our approach is that we apply FEM analysis directly to the image to obtain the result in the form of an image with which segmentation is made. In [21], they proposed a variational approach to image segmentation with a convex variational model and use FEM to numerically solve the problem. Although their work could be considered comparable to our approach, the principle underlying their segmentation method is completely different from ours. Thus, the authors of conventional methods use FEM, however their approaches are different from ours.

### Review of FEM [22]

**Figure 1:**
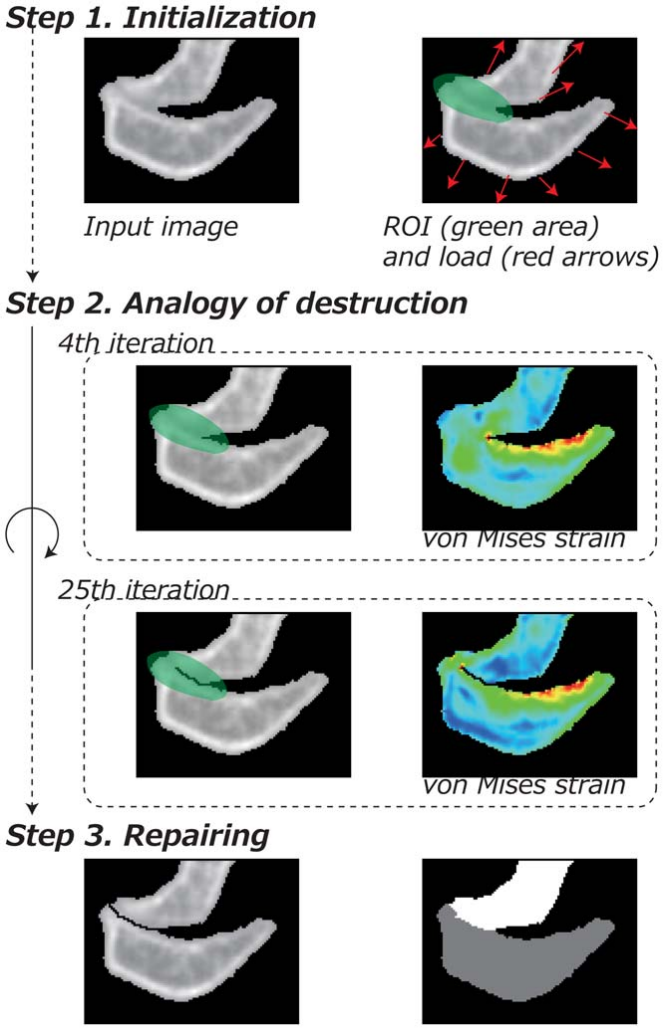
Flow chart of the proposed segmentation algorithm. Green area in the top and left middle are ROI and the red arrows show some representatives of loading forces. Images in the right and middle show distribution of von Mises strain and red pixels indicate high value. Using analogy of destruction we repeat removing these red pixels until the object gets separated along the interference region. In the bottom, the result of segmentation is shown where the removed pixels are salvaged to be included in the segments.

Our segmentation method is based on an idea that a structure can be broken along its structurally weak part which can be detected by computing *strain* distribution generated by applying some external load to the structure [22]. For instance, the top right image of Fig. 1 shows such loads in red arrows. Under such loading condition, the structure elastically deforms. The magnitude of the deformation varies place to place according to the shape of the structure and the material strength at the location. Such deformation can be measured as value called *strain* which represents local and net deformation (without rigid transformation) at each point of the structure. In the middle right images of Fig. 1 show the strain distribution under the loading condition. A physical structure breaks at the place with large strain which exceeds its limit of the material.

The strain distribution can be numerically computed using FEM by solving the static structural problem. In the following sections, we briefly review FEM and also introduce Pixel based FEM [23] which is an application of FEM directly to images. For more details about FEM, we refer the reader to such a text book as [22].

#### Finite elements

**Figure 2:**
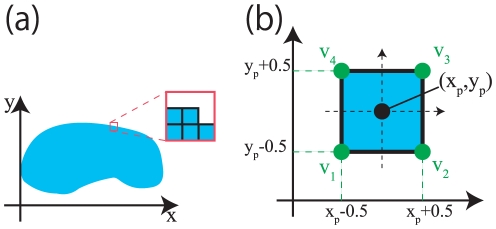
Node definition. (a) is Finite elements and (b) is Nodes of an finite element. The blue area in (a) is an object represented with a set of quadrilateral elements. (b) shows four corner nodes of an element.

In FEM, the structure is decomposed into a set of *finite elements*. Among various types of the finite elements we use quadrilateral elements as shown in Fig. 2. In this figure, (a) shows an object which is decomposed into a set of quadrilateral elements. For each element, four nodes (corners) 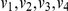 are defined as shown Fig. 2(b). By applying external force 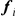 to the node 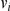, the deformation causes some displacement 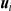 to 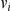. 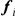 and 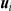 are a two dimensional vector of force and displacement respectively. A linear equation can be derived to represent the relationship between the displacement at these nodes and the forces:
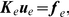
(1)where 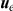 and 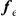 are defined as:
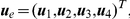


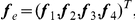


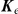
 is called *elemental stiffness matrix* that is constant for the element. The derivation of 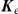 will be given later in this section.

#### Strain-displacement relationship

The application of external forces at those four nodes causes displacement 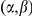 also at each point inside the element. And the strain 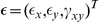 at this point is defined by derivatives of 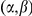 w.r.t. *x* and *y* as follows:
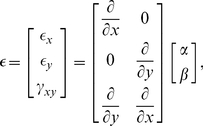
(2)where 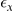 denotes the strain in the *x* direction, 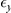 is that in the *y* direction, and 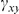 is the shear of strain.

The displacement 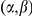 can be estimated by interpolating the displacements at the four nodes 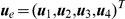. By using a bi-linear interpolation to estimate 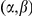 from 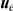, the strain can be represented by the following linear equation.
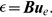
(3)The matrix 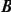 is called *strain-displacement matrix*.

#### Stress-strain relationship

The relationship between stress 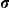 and strain 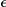 is obtained using the widely known Hooke's Law as follows:
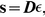
(4)

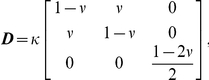


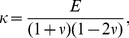
(5)where 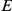 and 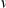 denote Young's modulus and Poisson's ratio, respectively.

#### Load-displacement relationship

Now we derive relationship between the external forces and the displacement by use of the principle of virtual work, that is, when a structure is in equilibrium, the internal work 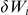 consorts with the outer work 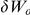 generated by applying arbitrary infinitesimal displacement to the structure. 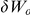 is represented by
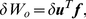
(6)where 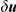 denotes virtual displacement, which is infinitesimal so as not to affect the external forces. The internal work 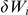 is given by integrating the product of virtual strain and stress over the finite element.
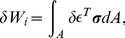
(7)where 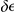 is virtual strain given by 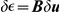. 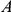 denotes the area of the finite element.

By the principle of virtual work 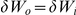, we obtain
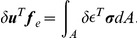
By substituting Eq. 3 and 4 to this equation, we obtain
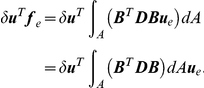
Since this equation holds for arbitrary virtual displacement and strain, the following equation of equilibrium is derived. It defines the relationship between the external forces and displacement at the nodes.
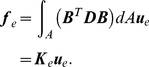
(8)This matrix 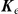 is equal to 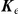 in Eq. 1.

#### System equation

By assembling the Eq. 8 for all the elements, we obtain a linear system equation to solve a static structural problem to find displacement of nodes for given external forces. We define node numbers 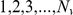 for the set of all the nodes 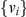, where 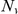 denotes the total number of nodes. We also define the node displacement vector 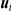 and external force vector 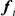 for the node 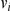. Then the total node displacement vector 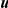 is defined by arranging 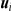 in this form:
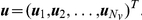
The total external force vector 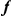 is also defined in the same way:
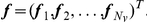
By using those vectors the system equation for the static structural problem can be represented by
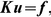
(9)where 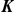 is called the *system stiffness matrix*.

#### von Mises strain

From the node displacement 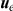, we can estimate strain vector (deformation ratio) 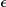 using Eq. 3. As the measure for the total amount of the strain, von Mises strain 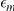 is used as a scalar expression of strain, which can be defined as
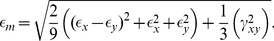
(10)We rewrite this equation using
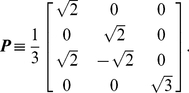
(11)Then we obtain
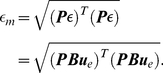
(12)Note that 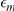 is defined for each element.

### Pixel based FEM

We apply FEM to CT image (a two-dimensional image). This approach was first proposed by Bendsoe and Kikuchi [23] as “Pixel based FEM.” The pixels of the image 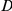 are squares aligned with an *xy* coordinate system in a reticular pattern. In this paper, we express pixels as 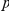 and its value as 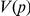. We consider a set of foreground pixels 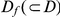 represents a structure. In this paper, we use a simple thresholding method to extract the foreground with a conservative threshold value so as not to miss vague foreground pixels. Although more sophisticated methods can be used here, we regard this problem out of scope of this paper as the choice of the method does not affect our method.

For each pixel of 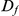 we define a quadrilateral element of FEM. The four corners of 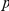 will be nodes of the element. From this point, pixels and elements are regarded as the same.

## Methods

**Figure 3:**
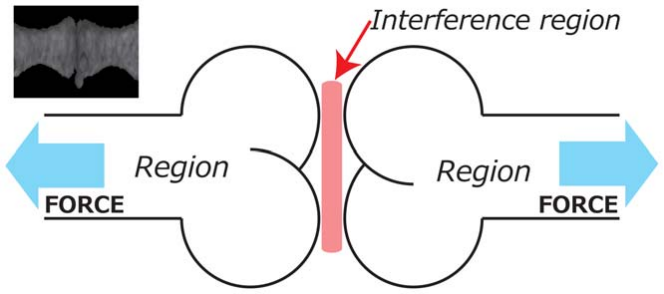
Schematic overview of the proposed segmentation method. On the upper left is a CT image of a joint of a mouse's tail which consists of bones and cartilage. We assume the bone as a region and the cartilage as an interference region and separate the regions in the interference region. By applying forces to the bones so as to generate high strain to destroy the interference region.

Here we propose a CT image region segmentation method using FEM in which boundary conditions are optimized to achieve superior image segmentation. We first discuss two-fraction partitioning problem. Fig. 3 illustrates the proposed approach to image segmentation. We assume that the target object has an *interference region* between *regions*. Since we usually use the word “interface” as the boundary between foreground and background. In order to avoid confusion, we named the area between regions as interference region.

The method is based on the intuitive observation that a structure can be broken along this interference region by applying appropriate external forces because it is structurally weaker than other regions and carries a higher level of strain. We thus find pixels with a higher level of strain and remove them to break the object. Strain can be computed using the FEM.

Fig. 1 gives a flow chart of the proposed segmentation method including three main steps. In the figure, a CT image of mouse's shoulder is shown as an example. We will discuss these steps in the following sections.

### Initialization

For applying FEM, we need to define material properties of Young's modulus 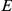 and Poisson's ratio 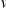. In the case of bone, 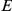 is related to the cube of its density [24]. Accordingly we assume that the Young's modulus 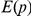 of pixel 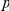 is proportional to 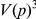, in this paper. Poisson's ratio 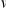 is set constantly as 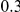.

Eq. 9 is a system of simultaneous linear equations. However, we cannot solve this equation, since 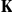 is a singular matrix. In general cases, to solve Eq. 9, a displacement constraint must be set to the nodes. This operation is equivalent to fix the nodes to the ground. However, selection of fixing nodes is not a trivial problem. Instead, we set weak springs to all nodes, and these springs are connected to the ground. Setting the spring coefficient as 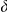, the stiffness matrix is modified as:
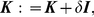
(13)where 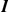 denotes the identify matrix. 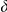 is not sensitive to computation, so we determine 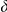 experimentally, which is sufficiently smaller than the largest element of 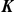.

**Figure 4:**
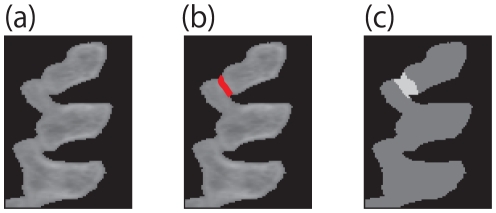
ROI setting. (a) is Input image 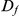, (b) is Interference region, and (c) is ROI 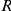. (a) is the input foreground image. The red line in (b) indicates an interference region. The white area in (c) is user specified ROI 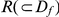.

We assume the user roughly knows the location of the interference region. The user must define a region of interest (ROI) 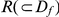 such that it includes the interference region. Fig. 4(a) shows an image 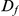, which is a 2D foreground CT image of a mouse's backbone. We want to segment along the interference region, which is highlighted as red in Fig. 4(b).

#### Loading condition setting

In order to find pixels to be removed with a higher level of strain, we evaluate von Mises strain 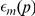 of each pixel 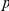 with the displacement of nodes 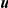, which is obtained by solving Eq. 9. For this purpose, we need to set the loading condition of external forces 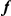. One method was to do it manually [1], but this is not a realistic way particularly for complex structures. In this paper we propose a method to automatically find some appropriate 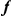 by casting this problem to an optimization problem.

A desirable loading condition creates high strain in the ROI. As von Mises strain is a non-negative scalar value, we set the objective function as
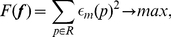
(14)where 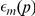 denotes the von Mises strain of element 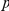.

#### Optimization

Gradient ascent method is used to calculate loading condition vector 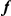.
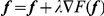


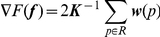


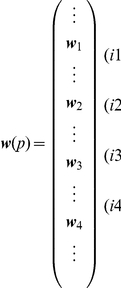


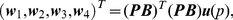
(15)where 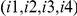 denote the four node numbers of element 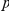 and 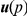 denotes the displacement vector of 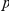 which is equal to 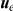 defined by Eq. 1. The other elements of 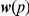 are all zero. This 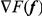 can be derived using Eqs. 9, 11, and 13. 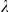 is the user input coefficient. Since 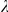 is sensitive to convergence speed, we use the Armijo rule [3] to set 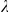.

**Figure 5:**
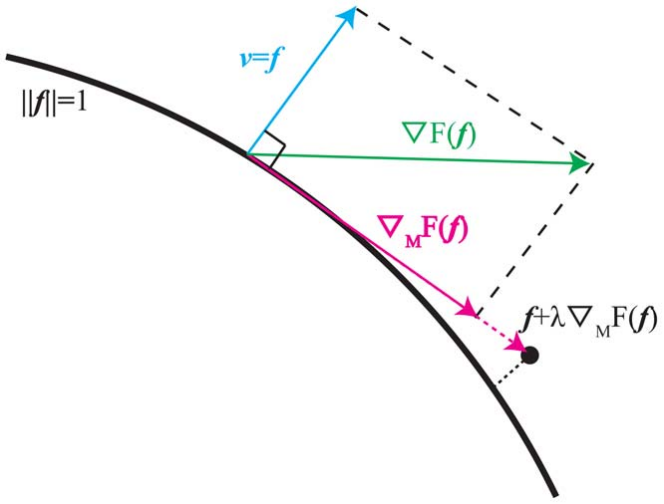
Loading condition on manifold surface. 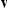
 is a normal vector of 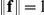 and is equal to 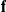.

We limit 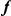 to the surface of 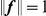. W need to modify 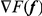 to be 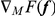 as shown in Fig. 5. Instead of Eq. 14, we use the following equations:
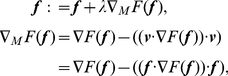
(16)where 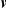 denotes the normal vector of 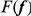 and is equal to 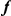.

We set external force only to the boundary nodes of 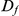. We set the initial loading condition vector 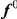 to obtain boundary nodes away from 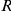 (Fig. 3) because the level of strain will be large when nodes around pixels are moved in different orientations. By calculating the center point 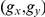 of 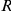 assuming the CT value as the weight, we set 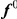 for the 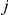-th node 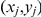 as 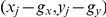. By aligning all the initial loading conditions of all boundary nodes and normalizing them, we make 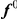.

**Figure 6:**
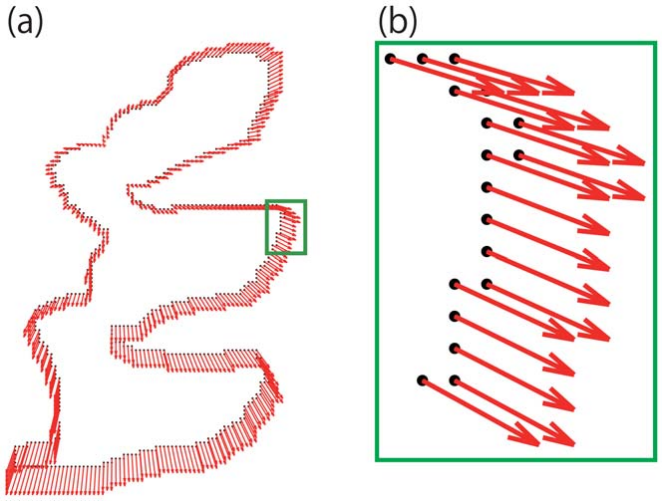
**Results** **of loading condition optimization.** (a) is Loading condition and (b) is Close up view of (a). The black points are boundary nodes, and the red arrows are loading condition vectors for each node. (b) is a close-up of (a) from the green area.

Fig. 6 shows the optimized loading condition vector for the image in Fig. 4(a) with ROI of Fig. 4(b). In Fig. 6, the black points are boundary nodes, and the red arrows are the loading condition vectors of the black points.

### Analogy of destruction

We imitate destruction by iterating the two steps of strain calculation and pixel removal. As previously outlined, 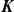 is taken as the foreground pixel set 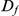. With 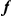, we calculate 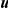 by solving Eq. 1, and calculate 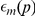 using Eq. 11.

The pixel 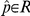 with the highest von Mises strain value is removed from 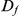 by setting it as a background pixel in this form:
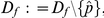
where
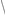
denotes set subtraction operator.

Then, we check if the topology (connectivity) of the foreground 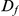 changes or not. When 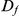 decomposes into multi sets, the iteration halts and the process progresses to the next section. Otherwise, we repeat the above strain calculation.

### Repairing

We label the remaining pixel sets in 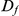 using neighbor relationships. And we apply the region growing method until all removed voxels are classified into one of the segmented regions.

### Expansion to multi-segmentation

Here, we expand the proposed segmentation method to form a multi-segmentation problem. Assuming that structurally weak areas should be segmented, a decomposition priority is calculated using the ROI strain.

**Figure 7:**
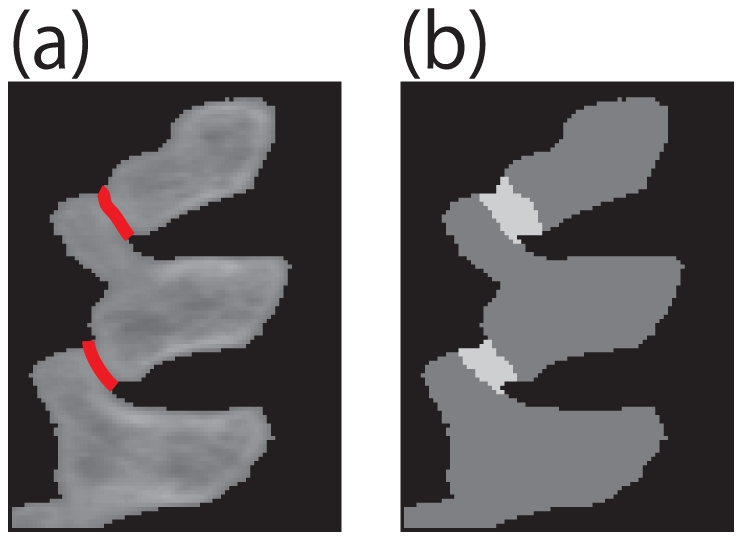
Expanding to multi segmentation. (a) is Interference regions and (b) is ROIs. In (a), red lines are the interference regions. In (b), two ROIs are shown in white.

In line with the above discussion on two-fraction partitioning, one ROI is set. Here, we consider a case with 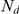 interference regions. The user sets 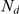 ROIs. Fig. 7(a) is the same sample as Fig. 4(a), however two interference regions (red lines) are considered and two ROIs (white regions) are specified in Fig. 7(b).

**Figure 8:**
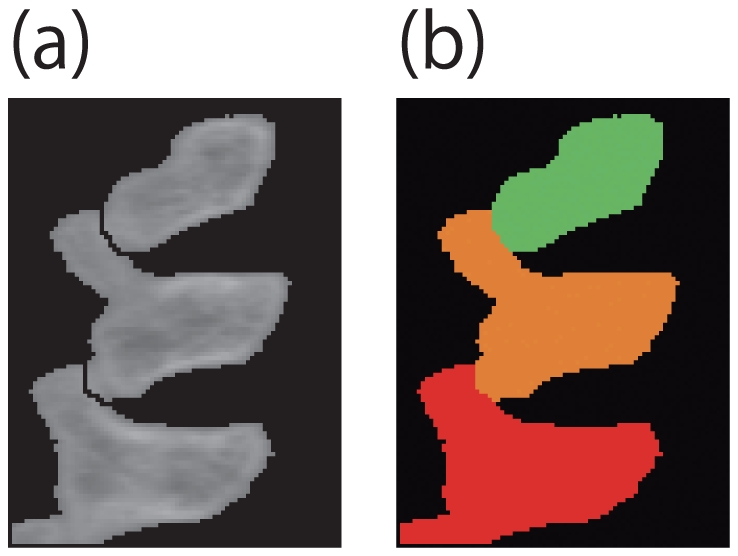
Result of **Fig. 4**. (a) is the result of destruction. (b) is the repaired version of (a).

We segment ROI one by one. In order to determine the iteration order, we calculate values of Eq. 13 for all ROIs and divide them by their area to evaluate their structural weakness. We iterate region segmentation for each of ROIs with the order of their structural weakness. The total number of iterations is 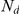. The result for the case of Fig. 7 is shown in Fig. 8.

### Doubtful ROI specification

**Figure 9:**
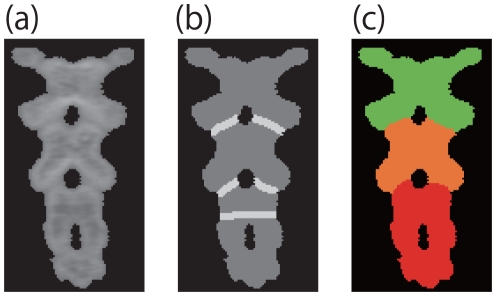
Backbone of a mouse (a) is the input CT image. The white areas in (b) are the ROIs. (c) is the result of the proposal method. Note that segmentation is not made along the lowest ROI in (b). Such ROIs are sometimes mistakenly defined for the region without interference. Our method can skip such incorrect ROIs.

The ROIs must be given by a user, however in some cases he/she makes a mistake to specify ROIs to regions that only look like interference but are not real ones. It is worth mentioning that we can call off the segmentation for those incorrect ROIs by using the structural weakness values as they are very low for such incorrect ROIs. It is demonstrated using the sample of Fig. 9 in the next section.

## Results

**Figure 10:**
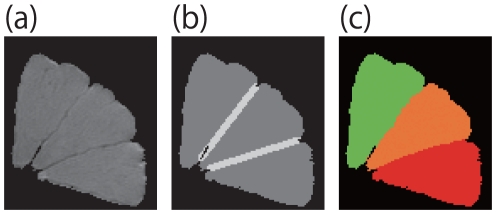
Sections of orange (a) is the input CT image. The white areas in (b) are the ROIs. (c) is the result of the proposed method.

**Figure 11:**
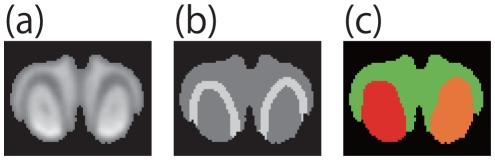
Front teeth of mouse. (a) is the input CT image. The white areas in (b) are the ROIs. (c) is the result of the proposed method.

We applied the proposed algorithm to a number of CT images. Figs. 9(a) 10(a), and 11(a) are CT images of parts of an orange, a mouse's backbone, and a mouse's front teeth, respectively. The ROIs are highlighted in white in Figs. 9(b), 10(b) and 11(b).

Table 1 shows the conditions and parameters of the experiments. We implemented our algorithm using C++ and performed experiments using a Windows 7 64-bit computer with an Intel(R) Core i7 CPU 920 @ 2.67 GHz and 12 G RAM.

**Table 1:** Conditions of experiment 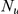 is the number of ROIs and 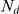 is the number of iteration.

	Size	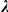	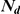	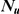
Fig. 1	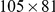	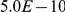	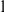	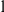
Fig. 4	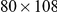	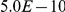	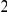	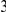
Fig. 10	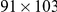	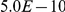	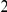	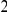
Fig. 9	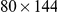	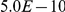	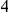	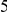
Fig. 11	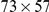	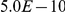	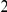	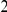

Figs. 9(c), 10(c) and 11(c) show the segmentation results of Figs. 9(a), 10(a) and 11(a), respectively. Table 2 shows the computation time necessary to obtain the experimental results.

**Table 2:** Computational times for step 1 and step 2.

	Step 1	Step 2	Total
Fig. 1	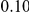	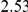	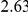
Fig. 4	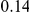	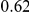	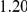
Fig. 10	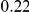	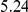	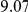
Fig. 9	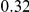	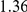	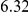
Fig. 11	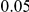	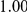	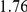

The values are computational times (min) for each step. Step 1 is for loading condition optimization, and step 2 is for destruction and repairing.

## Discussion

### Comparison with graph cut-based method

The graph cut method was originally proposed by [14] for image segmentation. A user defines hard constraints for segmentation by specifying two sets of pixels (seeds) that must be a part of the two segments. The other pixels are segmented into either one of these segments automatically by solving a global optimization problem. By regarding the image as a graph of pixels, the pixels are connected by arcs to their neighboring ones and also to two terminal nodes representing the two segments. Costs are defined for these arcs according to the similarities between their incident pixels. Then the max flow between the terminal nodes is computed so that the graph is cut into two parts along the max cut. These two graphs constitute the segmentation.

**Figure 12:**
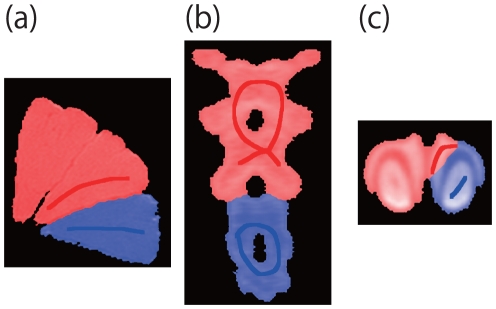
**Results** **of the graph cut method.** (a), (b) and (c) are the results of the graph cut method for the images of Fig. 10, Fig. 9, and Fig. 11, respectively. (a) and (b) are considered fine, but (c) is not an appropriate result.

In our experiments with the graph cut we use the same cost functions for all the images. The cost function between neighboring pixels is defined by using an exponential value of the difference between the CT values of these pixels. We conducted trials of segmentation to tune parameters involved in the cost function to give good results. As for the seeds of the graph cut, we also tested their several patterns and Figures 12(a), (b), and (c) are the ones resulting in the best segmentations.

In the case shown in Fig. 10(a), interference regions are thin and clear. It is easy to segment this type as shown in Fig. 12(a). Although this is a CT image of an orange rather than a skeleton, Fig. 10(c) also shows successful segmentation. From these results, our algorithm works well in the case of clear CT image, which could be segmented by using conventional method.

Here we also discuss the cases of Fig. 9(a) with multiple interference regions. As these interference regions are not ambiguous, the graph cut method produces successful results as shown in Fig. 12(b). The proposed algorithm also works well. Additionally, although Fig. 9(b) contains erroneous ROI, the proposed algorithm avoids segmentation in this region. From these results, our algorithm can avoid wrong segmentation when the user knows the number of the regions.

In the case of Fig. 11(a), whose interference region is clear but comparatively large. Because of its size, the energy of the region is the same as that of the interference region, and Fig. 12(c) shows the drawback of using the graph cut method in this case. From these results, our algorithm also works in the case of CT image with ambiguous interference region or wide interference region.

### Influence of ROI

**Figure 13:**
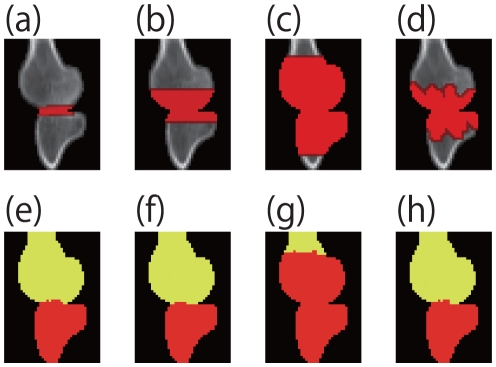
**Results** **of ROI test.** Images in the top row are input CT images with ROIs (red regions). Images in the bottom row are segmented results for corresponding images in the top row.

We tested a number of ROI patterns as shown in Fig. 13, which is an image of a human knee (The Volume Library [25])(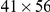 pixels). Fig. 13(a) shows the input image with a standard ROI (highlighted in red), and Fig. 13(e) is the segmentation result for Fig. 13(a). Figs. 13(b), (c), and (d) are also input images with a large ROI, a very large ROI and an irregularly shaped ROI, respectively. Figs. 13(f), (g), and (h) are the results of segmentation for Figs. 13(b), (c), and (d), respectively.

Figs. 13(b) and (d) are similar to Fig. 13(a). This indicates that the area and shape of the ROIs are not dominant with the proposed algorithm. However, failed segmentation is shown in Fig. 13(g), whose ROI is almost the same size as the entire foreground of the input image. These experiments indicate that it is necessary to set an appropriate ROI because images generally contain more than one structurally weak areas. In other words, if the user sets ROIs based on a certain level of prior knowledge, successful segmentation can be guaranteed.

### Computational time

Table 2 shows computational times required for segmentation. The proposed algorithm is comparatively slow because FEM was used for structural analysis and iterated. The computational time taken for FEM depends on the size of 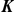. The total iteration time is similar to that of area 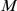 in the interference region. Thus, the proposed algorithm's total time complexity can be estimated as
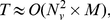
(17)where 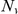 is the number of foreground vertices (nodes).

### Shape limitation

Since our algorithm is designed to segment joints of a skeleton, it prefers such objects with relatively high aspect ratio around the joints as shown in Figs. 9(c), 10(c) and 11(c). However, it is not suitable for objects with isotropic shapes which tend to have uniform distribution of strain.

### Conclusion

We proposed a CT image segmentation method using structural analysis aiming at segmenting a CT image of a skeleton into pieces of the bones at their joints where the contrast of the image is usually weak. In our novel approach, we compute strain distribution of a structure in the image to find those pixels with high level of the strain then segment the image along these pixels. To compute the strain distribution, we use Pixel based FEM method. In order to automatize the procedure, we introduce an optimization method to derive loading condition (external forces) so as to bring high strain to the pixels in user defined ROI. From experimental results, it can be concluded that the proposed algorithm works well with CT images with large or ambiguous interference regions. Our future work includes extension to three dimensional problems and improvement of computational efficiency.
